# Parents’ health information seeking behaviour – does the child’s health status play a role?

**DOI:** 10.1186/s12875-020-01342-3

**Published:** 2020-12-10

**Authors:** Isabel Baumann, Rebecca Jaks, Dominik Robin, Sibylle Juvalta, Julia Dratva

**Affiliations:** 1 Institute of Health Sciences, School of Health Professions, Zurich University of Applied Sciences, Winterthur, Switzerland; 2 Medical Faculty, University of Basel, Klingelbergstrasse 61, Basel, 4056 Switzerland

**Keywords:** Parents, Health information, Information seeking behaviour, Digital media use, Illness, Disability

## Abstract

**Background:**

Digital media are increasingly abundant providing a wide scope of health information. To date, very little is known about parental health information seeking behaviour for child health outside of English-speaking and Nordic countries. Our study “Digital parental counsellors” examines how parents search for health information in digital media, print media and among “personal contacts”, distinguishing between the search for information about general child health and development and child’s acute illness, and comparing information seeking behaviour by disability status of the child.

**Methods:**

The population-based sample consisted of 769 parents with children aged 0–2 in the German-speaking region of Switzerland returning the study questionnaire (30% response rate). We developed a frequency score of use of different information sources and conducted bivariate and multivariate linear regression analyses to describe parental search behaviour and the association with child’s disability status.

**Results:**

The sample consists of 88% mothers (mean age: 35.7 years SD 4.33). Children’s mean age is 16 months (SD 7.1), 49% of the children are female and 6% have a disability. Parents use digital media significantly more frequently to search for information about general health and development questions than about an acute child’s illness (*p* < 0.001). In case of acute child’s illness, parents refer to their paediatrician, family members and other personal contacts significantly more frequently than other information sources (*p* < 0.001). The use of digital media and “personal contacts” does not significantly vary between parents with and without a disabled child, whereas the use of print media does (*p* < 0.02). Moreover, irrespective of disability, 45% of parents resort to the Internet prior to a paediatric visit and 27% after a visit when a visit did not answer all questions.

**Conclusions:**

Despite the high prevalence of digital media, personal contacts are still the most frequent health information resource for parents with young children, irrespective of the child’s health. Parents combine all information resources (online, print, personal network) to improve their understanding or check the validity of information received regarding their child’s health. It is thus of utmost importance, that the increasingly accessed digital information parents search for is correct, understandable and addresses parent’s concerns.

**Trial registration:**

BASEC Req-2017-00817 (30 October 2017).

**Supplementary Information:**

The online version contains supplementary material available at 10.1186/s12875-020-01342-3.

## Background

Today’s generation of young parents mostly grew up with digital media, regularly using them for information, communication, and networking purposes. Therefore, it is highly likely that they also use digital media in their role as parents. Literature indicates that the Internet has become increasingly important as a source of information for child health [1]. Moreover, the number of online portals developed to meet the needs of parents has greatly increased, offering information on a wide variety of topics such as deliveries, breast-feeding, and children’s diseases [2]. O’Connor and Madge [3] argue that the shift to the Internet is due to parent’s appreciation of its 24-h availability. Other reasons may be the increasing geographical mobility or the possibility to communicate anonymously in forums [3]. Another line of argumentation is the stress and uncertainty to which parents are exposed in everyday life such as compatibility of work and family [4]. On the one hand, the additional information that the Internet offers can be perceived as a supporting factor [5] or an opportunity to play an active role in decision-making in the sense of empowerment [6], while on the other hand the flood of information on digital networks and websites about child’s illness can also be overwhelming [7, 8], even frightening [9]. Indeed, digital information sources differ considerably in their reliability [10–12]. The capacity to assess trustworthiness of the content requires a high level of education as well as health and digital literacy [13, 14].

While the use of digital media seems to be widespread for searches about general information on a child’s health, nutrition and care [15], the question arises of how parents behave when their child has an acute illness or a long-term or permanent disability. There are hardly any studies on parental digital health information behaviour for Switzerland, and none of them directly addresses parental use of information resources in different child health contexts [16–18]. Most literature on parents’ digital media use originates in Anglophone countries and Scandinavia [19]. The few international studies focusing on either child’s illness or disability usually do not provide any comparisons with parents with healthy children.

A study by Walsh et al. [20] shows that parents whose children suffer from high fever seek advice from doctors, books or other parents, but less often from the Internet, while Yardi et al. found parents of a child with a disability to access the internet more frequently than parents of healthy children [21]. A representative study among adults in Germany has shown that those with multiple chronic conditions preferred a general practitioner as an information source compared with those without chronic conditions [2]. Parents with a disabled child may thus experience a need for specific health information. Previous research has shown that for some types of disabilities, information resources such as books are scarce [22–24]. For this reason, parents report to turn to the Internet to access additional or complementary information [25, 26]. Further, a series of studies has shown that web-based support groups provide parents with important practical and emotional support [23], particularly if the child’s disability is rare and other parents with similar concerns are difficult to find in a proximate location [22, 23].

Our study “Digital parental counsellors” (in German: “Digitale Elternratgeber”) examines parents’ patterns use of information resources (digital media, print media and personal contacts) in a population-based study sample with respect to two different health information seeking targets (information on general health and development vs. acute child’s illness), in the specific context of paediatric visits, and compares parents with children with and without a disability.

## Methods

### Study population

The study population consists of a population-based sample of parents with children aged 0–2 years in the German-speaking part of Switzerland. The birth registries of the City of Zurich and small municipalities in the same region randomly selected 2573 mothers who had given birth in the past 24 months and provided their names and addresses. Urban and rural municipalities were included to represent the urban/rural distribution of Switzerland (75%/25%).

Between January and May 2018 parents received an invitation letter with a link to an online questionnaire; together with the second reminder letter a paper questionnaire and a prepaid envelope were provided. Overall, 842 individuals responded to the survey of which 73 had to be deleted in the data cleaning process. Reasons for exclusion were empty questionnaire (*N* = 31), missing answers to key questions (*N* = 40), non-plausibility of key questions (*N* = 1), and double entry (*N* = 1).

A total of 769 questionnaires were completed, which represents a response rate of 30%. Four hundred twenty-nine participants (56%) responded online, 340 (44%) on paper. Samples were compared regarding the general use of digital media for information on child and adolescent health, main outcome, and the percentage of children with disability, as well as socio-demographic variables. Samples differed significantly by age, gender and socio-economic status, but not in the main outcome variable nor proportion of children with disability (Supplemental Table 1). Online and paper samples were merged for this analysis.

The cantonal ethical commission Zürich, responsible for the caption area in which this study was performed, confirmed the exemption from a full ethics review based on the Swiss Federal Act on Research involving Human Beings (Business Administration System for Ethics Committees (BASEC) Req-2017-00817), due to the fully anonymized data collection and anonymized health-related data.

### Questionnaire

The questionnaire covered different topics, from socio-demographic and health status of survey participant and child, the use of information resources for child general development and acute child’s illness, health information seeking, as well as e-health literacy and attitudes towards online health information of survey participant (not included in this analysis). The questionnaire was developed for this survey. Socio-demographic and health questions were taken from previous surveys on child health (Swiss infant feeding Study, https://www.zhaw.ch/de/gesundheit/institute-zentren/igw/forschung/kinder-und-jugend-public-health/; German Health Interview and Examination Survey for Children and Adolescents, KiGGS [27]), as well as newly constructed based on literature and paediatric consultation. E-health literacy was measured with the eHealth Literacy Scale (eHEALS) [28, 29]. Digital information seeking, trust and understanding questions are based on survey items from the Flash Eurobarometer 404 on European citizens’ digital health literacy [30] and Wainstein et al. (6)), respectively. The full questionnaire included 68 questions or items (see Harvard Dataverse: 10.7910/DVN/JI9GIJ.).

### Measures

#### Health-information seeking behaviour

Our main outcome of interest is the frequency at which parents use the information resources “digital media” for two different targets: either for seeking information on “general health and development” or on an “acute child’s illness”. Digital media presented in the questionnaire were social media (e.g. Facebook), websites for parents, apps on mobile devices, search engines, websites of paediatricians or children’s hospitals and official websites of health services or health organizations, and an “other” option. Open responses were checked it they could be grouped under one of the listed options, which was the case in all 32 “other” digital sources. Secondary outcomes are the frequency at which parents use the information resources “print media” (books, journals, newspapers and other print media) and “personal contacts”. The latter category included different persons and consultant, with whom a direct, personal contact and response to the individual need is assumed: paediatrician, other health professional, telephone consultation of a children’s emergency service or a children’s hospital, telephone consultation of a health insurance, family members and friends, acquaintances or neighbours.

Frequency scores for digital media summarize all six items, for print media all four items and for personal contact all six items mentioned above. Each item had five response options: never; rarely; sometimes; frequently; very frequently coded 0 (never) to 4 (very frequently). To calculate the sum scores, responses by type of resource were added and standardized to a scale from 1 to 100 to allow a comparison of the frequency between the three information resources.

Parents were further questioned if they had searched for health information before or after their last visit to the paediatrician and if yes, for which reasons. A list of reasons was proposed: I have received too little information from the paediatrician; information from the paediatrician was incomprehensible or contradictory; I needed to check information from the paediatrician; I had the need to exchange with others or to search for experiences and tips; I was looking for other therapies. For each, parents could answer: does not apply at all; does slightly apply; does partly apply; does apply; does apply very much. We created a binary variable summarizing the last three options into the category “does apply” and the first two into “does not apply”.

#### Population characteristics

Child characteristics: Child’s age is reported in months, sex as a binary variable and birthweight in grams. Gestational age is reported in weeks and parity indicates whether a child is the first born from her/his parents. Disability was defined as a binary variable based on the parents’ reporting of either a physical impairment (e.g. malformation), developmental delay, hearing or visual impairment or congenital disability.

Respondent characteristics: We distinguish between three roles of the respondents: Mother, father and other. In the regression analysis, a binary measure for parent’s role is used, excluding “other” respondents (*n* = 4). Respondents’ age is reported in years. Education is reported as no or compulsory education (max. 9 years of education), upper secondary education (e.g. apprenticeship or high school degree) or tertiary education (university or similar degree). Net household income is measured in five categories: less than 4500 Swiss francs (CHF); CHF 4500–6000; CHF 6000–9000; more than CHF 9000 and no indication / don’t know. Citizenship is used as a binary variable distinguishing between Swiss and Non-Swiss. Parents reporting a double citizenship were categorized as Swiss.

### Data analysis

First, we ran a descriptive analysis for the overall sample as well asd stratified by the child’s disability status (chi^2^-tests for categorical variables and independent-samples t-tests for numerical variables). Second, in case of the health information seeking target “acute child’s illness”, we further investigated the time point of the digital health-seeking behaviour and the reasons for doing so by disability, applying chi^2^-test. Third, we examined the standardized frequencies by type of health information seeking target in parents who provided information on both targets. For each information resource (digital media, print media and “personal contacts”), we compared the frequency of use for general health and development and acute child’s illness by applying box-plot analyses and paired-samples t-tests. We also reported the frequency of single items of information resources used by type of health-seeking target (independent-samples t-test) and disability status (paired-samples t-tests).

Fourth, we carried out ordinary least square (OLS) regression analyses for the primary and secondary outcomes for both health information seeking targets. The following confounders were included in the model: child’s sex, child’s age, parental age, parental sex and parental education.

We ran three sensitivity analyses with and without the following variables: First, due to many missing values for parents’ age, we excluded this variable. Second, we included net household income. Third, sensitivity analyses on the same models but on a sample restricted to parents who had answered the survey questions about both health information seeking targets instead of only one (*N* = 480 for digital media; 419 for print media; 597 for personal contacts). All analyses are carried out with the statistical software STATA/SE 15.1. Statistical significance was established at *P* < 0.01, to take account of potential multiple comparisons.

## Results

### Study participants

Our sample consists of 88% mothers (*n* = 673), the mean parental age is 35.7 years (SD 4.3), 76% have a tertiary level of education (*n* = 577), 72% are of Swiss nationality (*n* = 451) and 42% of the sample have a monthly net household income of over CHF 9000 (≈ € 8400, ≈ $ 9300, *n* = 305). Children’s mean age is about 16 months (SD 7.1), 49% of the children are female and in 6% parents report their child to have a disability. Study sample characteristics do not significantly differ by disability status.

### Frequency of use of information resources

The study sample shows a high use of all information resources (Table 1). Examining the frequency of use of information resources by the child’s disability status, we find significant differences between parents with a child with and without disability in use of print media. The former resort to print media significantly less frequently than the latter (*p* < 0.05), both when seeking information on general health and development and in the case of an acute child’s illness.

**Table 1:** Standardized frequency score of information resources used, stratified by health information seeking target and disability status

	Overall	Disability status		***P***-value
		Disability	No disability	
	*n* = 765	*n* = 42 (5.5%)	*n* = 723 (94.5%)	
Information resource: digital media				
General health and development, mean (SD)	33.0 (15.3)	33.7 (16.5)	33.0 (15.2)	0.8
Acute child’s illness, mean (SD)	28.6 (27.4)	26.71 (14.2)	28.75 (15.6)	0.5
Information resource: print media				
General health and development, mean (SD)	22.3 (16.8)	16.3 (13.1)	22.6 (17.0)	0.02
Acute child’s illness, mean (SD)	14.7 (13.7)	8.9 (7.5)	15.0 (13.9)	0.02
Information resource: personal contacts				
General health and development, mean (SD)	38.0 (12.5)	37.9 (10.4)	38.0 (13.0)	0.9
Acute child’s illness, mean (SD)	37.9 (14.8)	38.0 (13.4)	37.9 (14.9)	1.0

The *p*-value indicates the result from paired-samples t-tests. The standardized frequency is obtained by summarizing the reported frequency of use (never, rarely, sometimes, frequency, very frequently) and then standardizing the sum to a scale from 1 to 100 to make the scores comparable

### Use of information resources for different health information seeking targets

**Fig. 1:**
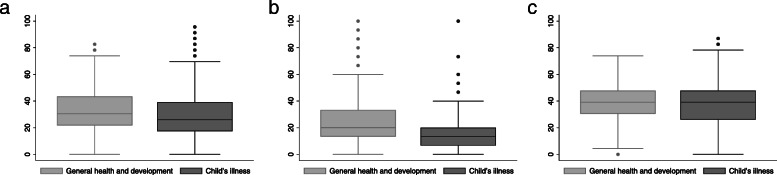
Use of information resources for different health information seeking targets. (**a**) Search for information in digital media. (**b**) Search for information in print media. (**c**) Search for information among “personal contacts”. Note: Respective N by information source: (**a**) *n* = 585. (**b**) *n* = 418. (**c**) *n* = 744. Difference across frequency scores: paired-samples t-test (**a**) *p* < 0.001, (**b**) *p* < 0.001, (**c**) *p* = 0.67

We find that parents use digital media significantly more often to search for information about general health and development questions (30.4, IQR 21.7–43.5) than for an acute child’s illness (26.1, IQR 17.4–39.1, *p* < 0.001) (Fig. 1a). Similarly, the median standardized frequency for print media use is higher when parents inform themselves about general health and development questions (20.0, IQR 13.3–33.3) than about an acute child’s illness (13.3, IQR 6.7–20.0, *p* < 0.001) (Fig. 1b). No difference by health information seeking targets is seen with respect to “personal contacts”: The median standardized frequency is 39.1 for both, general health and development questions (IQR 30.4–47.8) and an acute child’s illness (IQR 26.1–47.8, *p* = 0.67) (Fig. 1c).

### Use of individual items of information resources

Examining the individual information sources and their single items by type of health information seeking targets in Table 2, we receive a more differentiated picture of the results presented in Fig. 1a-c, even though numbers for single items were too small to compare by disability status. Whereas Fig. 1a indicates a significantly higher use of digital media for general health and development questions than for an acute child’s illness, results presented in Table 2 show that the difference is mainly driven by more frequent use of social media (*p* < 0.1) and of websites for parents (*p* < 0.05).

**Table 2:** Proportion of frequently or very frequently used items of the three information resources by health information seeking target

	Health information seeking target (n, (%))		***P***-value	Overall use (n)
	General health and development	Acute child’s illness		
Digital media				
Social media	31 (5.37%)	22 (3.81%)	0.09	577
Websites for parents	272 (50.28%)	236 (43.62%)	0.002	541
Apps on mobile devices	40 (7.59%)	32 (6.07%)	0.21	527
Search engines	342 (59.07%)	336 (58.03%)	0.59	579
Websites of paediatricians or children’s hospitals	71 (20.94%)	69 (20.35%)	0.76	339
Official websites of health services or health organizations	81 (13.99%)	72 (12.44%)	0.19	579
Print media				
Books	228 (30.94%)	148 (19.95%)	< 0.001	737
Journals	52 (7.19%)	24 (3.32%)	< 0.001	723
Newspapers	19 (2.64%)	8 (1.11%)	0.004	721
Other print media	35 (6.85%)	16 (3.13%)	< 0.001	511
Personal contacts				
Paediatrician	241 (32.61%)	419 (56.70%)	< 0.001	739
Other health professionals	139 (19.50%)	145 (20.34%)	0.57	713
Telephone consultation of a children’s emergency service	30 (4.19%)	69 (9.64%)	< 0.010	716
Telephone consultation of a health insurance	23 (3.25%)	48 (6.78%)	< 0.001	708
Family members	442 (60.14%)	391 (53.20%)	< 0.001	735
Friends, acquaintances or neighbours	363 (50.07%)	269 (37.10%)	< 0.001	725

We report the proportion of respondents who indicated to use an item frequently or very frequently among participants who reported using the item (overall use). The *p* value is identified using a paired-samples t-test

For print media, Table 2 indicates significant differences between the health information seeking targets for all items: books (*p* < 0.001), journals (*p* < 0.001), newspaper (*p* < 0.01) and other print media (*p* < 0.001). For all items, the frequency is higher for general health and development questions than for an acute child’s illness.

We also find significant differences between most items of “personal contacts”: paediatrician, telephone consultation of a children’s emergency service, telephone consultation of a health insurance, family members and friends, acquaintances or neighbours (all *p* < 0.001) by the health information seeking target. The paediatrician, telephone consultation of a children’s emergency service and telephone consultation of a health insurance are used more frequently in the case of an acute child’s illness. In contrast, family members and friends, acquaintances or neighbours are contacted more frequently for questions about general health and development.

### Use of digital media before and after a paediatrician visit

Parents were asked whether they used digital media to access health information *before* or *after* their last visit to the paediatrician. Almost half of the parents (45.5%, *n* = 195) did so *before* the visit while 27.1% did so *after* the visit (*n* = 117). A chi2-test reveals that differences between parents with a disabled and a non-disabled child are not statistically significant.

Among parents searching for information *before* the visit inform themselves about the health issue in general (90%), as well as about alternative medical (43%) and academic medical treatment options (47%). When asked for the reasons why they use digital media *after* the visit to the paediatrician, slightly more than 54% of the parents had the need to exchange with others or to search for experiences and tips, about 39% were looking for other therapies, about 38% needed to check information from the paediatrician, about 28% indicate to have received too little information and, for about 17%, the information was incomprehensible or contradictory. Differences between parents with a disabled and a non-disabled child are not statistically significant.

### Health information seeking behaviour by disability status

In Table 3, we present the results from the ordinary least square (OLS) regression analyses. The main analyses (models 1 and 2) do not yield significant association between the frequency of parents’ use of digital media and disability, neither in case of general health and development questions nor in case of an acute child’s illness. With respect to model covariates, we find significant effects for the child’s age (β-Coef.: -0.21, CI: − 0.41-0.02) in the general health and development model (1). In both models we find an effect for parents’ education: parents with compulsory education, only, use digital media much more frequently than parents with upper secondary education in the general health and development model (1) (β-Coef: 10.58, CI: 1.24–19.92) and in the acute child’s illness model (2) (β-Coef: 12.61, CI: 0.80–24.43).

**Table 3:** Association between parents' health information seeking behaviour and child's disability (multivariate ordinary least square regression (OLS))

Variables	***Digital media***		***Print media***		***Personal contacts***	
	(1) ***GHD*** *n = 564*	(2) ***CHI*** *n = 491*	(3) ***GHD*** *n = 567*	(4) ***CHI*** *n = 421*	(5) ***GHD*** *n = 612*	(6) ***CHI*** *n = 598*
	*β-*Coef. [95% CI]	*β-*Coef. [95% CI]	*β-*Coef. [95% CI]	*β-*Coef. [95% CI]	*β-*Coef. [95% CI]	*β-*Coef. [95% CI]
Child’s characteristics						
Disability (ref. no disability)	−1.13 [− 6.54; 4.23]	−2.46 [− 8.06; 3.14]	−8.32 [− 13.90; − 2.73]	−6.30 [− 10.16; − 2.44]	0.72 [− 3.60; 5.05]	0.44 [− 4.63; 5.50]
Female (ref. male)	−1.11 [− 3.62; 1.41]	0.92 [− 1.84; 3.69]	1.88 [− 0.99; 4.75]	0.16 [−2.54; 2.87]	0.20 [− 1.79; 2.18]	0.18 [− 2.18; 2.54]
Age	− 0.21 [− 0.41; 0.02]	−0.13 [− 0.34; 0.08]	0.05 [− 0.15; 0.25]	0.15 [− 0.05; 0.35]	−0.06 [− 0.20; 0.08]	−0.12 [− 0.30; 0.05]
Respondent’s characteristics						
Age	−0.06 [− 0.35; 0.23]	−0.09 [− 0.43; 0.26]	−0.32 [− 0.65;0.01]	0.09 [− 0.21; 0.39]	−0.03 [− 0.28; 0.21]	−0.02 [− 0.30; 0.26]
Mother (ref. father)	1.83 [− 2.51; 6.17]	−1.81 [− 6.10; 2.47]	−3.59 [− 7.82; 0.64]	2.54 [− 1.68; 6.76]	− 2.30 [− 5.52; 0.93]	− 4.31 [− 8.01; 0.61]
Education (ref. upper secondary education)						
No or compulsory education	10.58 [1.24; 19.92]	12.61 [0.80; 24.43]	8.65 [−2.04; 19.35]	7.40 [− 4.70; 19.50]	5.57 [0.35; 10.78]	5.31 [− 0.57; 11.19]
Tertiary education	1.68 [− 1.57; 4.94]	2.00 [− 1.66; 5.66]	0.30 [− 3.18; 3.79]	−1.69 [− 5.05; 1.67]	0.26 [− 2.23; 2.74]	1.07 [− 1.93; 4.07]
Constant	37.45 [24.58; 50–32]	32.42 [17.63; 47.21]	33.36 [18.97; 47.74]	10.24 [− 2.58; 23.07]	40.75 [30.19; 51.31]	41.02 [28.92; 53.12]

*GHD* general health and development question, *CHI* acute child’s illness

With respect to parents’ use of print media we observe a statistically significant association for the child’s disability both for general health and development questions (model 3) (β-Coef: -8.32, CI: − 13.90 - -2.73) and an acute child’s illness (model 4) (β-Coef: -6.30, CI:-10.16 - -2.44) while we do not find significant results for use of “personal contacts” (models 5 and 6). However, the model covariate parental education is significantly associated with information seeking: parents with no or compulsory education address “personal contacts” much more frequently for information about general health (model 5) (β-Coef: 5.57, CI: 0.35–10.78) and development and acute child’s illness (model 6) (β-Coef: 5.31, CI: − 0.57-11.19) than parents with an upper secondary level of education. In addition, in model (6) we find an effect for respondent’s role: mothers have a significantly less frequent use of personal contacts in case of an acute child’s illness than fathers (β-Coef: -4.31, CI: − 8.01 - 0.61).

Sensitivity analyses excluding the covariate parents’ age yielded consistent results for the primary outcome variable. In sensitivity analyses including net household income, the income variable did not prove to be a significant covariate, however, its inclusion lead to a loss of the statistical significance of education. Moreover, it contained a large number of missing values. Sensitivity analyses including only those observations for which information was reported for both general health and development and an acute child’s illness, lead to a lower number of observations for each analysis with no relevance for the effect estimates. We therefore present the results of the initial models.

## Discussion

Albeit an increase in digital media use, “personal contacts” remain the most frequently used information resource for parents with small children, both for questions on general health and development as well as on child’s acute illness. Nevertheless, parents query digital media, e.g. before and after the doctor’s visit to inform themselves. Print media were the least frequented medium, and significantly less used by parent’s whose child had a disability.

These findings may be explained by the media richness theory suggesting that more complex information is best conveyed through a “richer” medium [31]. “Personal contacts” in most cases imply a face-to-face communication or a directed and immediate interaction with a person which does not only provide parents with an immediate feedback, but also with abundant and more detailed verbal and non-verbal information, and it might therefore be a richer medium in the circumstances under investigation. Especially, when the advice and guidance requested concerns a particular condition and symptoms, such as in case of an acute or chronic illness or disability. At the same time, digital media may be considered a “poorer” medium due to a lack of non-verbal gestures, cues and signs and limited verbal cues because of technical limitations [32]. Social media, possibly the richest digital medium with respect to interaction and fast feedback, was only used by 5% of digital users in this study sample [15]. Evidence on adaption of media richness theory in connection to social media focusing on challenges as well as opportunities are still sparse [33]. The use of either digital media or “personal contacts” does not significantly vary between the information seeking targets, general health and development and acute child’s illness. Comparable results were found in a study on Norwegian mothers [34].

When examining the single items of “personal contacts”, our study shows that in the case of an acute child’s illness paediatricians are contacted most frequently, while family members and friends, acquaintances and neighbours come second and third, respectively. This finding is in line with earlier studies showing that “personal contacts” – especially the paediatrician – are of particular importance to parents in case of an acute child’s illness [20, 35], this may be explained by the urgency often associated with an acute illness, as well as the need of treatment, e.g. prescriptions by a paediatrician. Similarly, other studies on health information seeking behaviour have shown that individuals with serious health conditions tend to use health care providers as primary sources of information [36].

Interestingly, for both health information seeking targets, parents of children with a disability use print media less frequently than parents with a non-disabled child, while the other information sources are used similarly. One potential explanation may be that specific print literature for certain disabilities for lay persons is missing or quickly outdated, and the information need often is particular to the childs’ condition. Porter and Edirippulige reported that this was in fact the case with online-availability of health information about children with cochlear implants in 2008 [25]. Yet, unlike Porter and Edirippulige, we do not observe a differential use of digital media. Possibly, the digital contents have improved in the past 10 years and it seems reasonable to assume that parents now seem to find more information online. Moreover, for parents with a disabled child it may be less time-consuming to browse information online than to sit down to read print media [23].

A considerable proportion of parents used digital media to search for health information *before* or *after* their last visit to the paediatrician due to an acute child’s illness. Among these parents, searching for digital information *before* the visit to the paediatrician is more common than *afterwards*. The main reason was to read up on symptoms and relevance of the illness, as well as on treatment options prior to talking with the paediatrician. This finding supports earlier results showing that searching for information *before* the visit may help parents to be more confident in the discussion with the paediatrician about their child’s health [37]. *After* the visit, exchange with others or the search for experiences and tips is the most frequent reason for using digital media. Some parents, however, state that they had not received enough or understandable information or felt the need to check some facts, indicating that parents rely on the digital resources to counter lack or insufficient communication. Parents of a child with a disability do not significantly differ from those with a child without disability in terms of their use of digital media *before* or *after* their last visit to the paediatrician.

A strength of our study is the recruitment a population-based target group of parents with children aged 0–2 years and investigating different health information seeking situations. Having provided both online and paper-questionnaires ensured participation of both digitally affine as well as less affine parents, in fact we did reach parents who rarely used or were hesitant about informing themselves digitally [6]. However, we cannot fully exclude having reached a more digitally literate or experienced population with our survey, limiting a generalization of our results to parents in general. It was assumed that by providing a paper questionnaire, we might also be able to recruit more low-educated parents than with the online questionnaire alone [15]. Indeed, the paper sample was filled out more often by parents with a lower education level and lower income resulting in a higher representativity of the sample. However, we observe that overall individuals with a tertiary education are overrepresented in our responding sample. While slightly more than a third of the Swiss population below 44 years has a tertiary degree [38], it is three quarters in our sample.

Although the items in the instrument were adapted from other established instruments, no reliability or validity data are available for the investigator-created instrument. Further psychometric evaluation of the instrument should be conducted before additional use in future studies.

## Conclusion

Our study shows that despite the wide-spread use of digital media, “personal contacts” are still the most frequented source of health information for parents, irrespective of a child’s disability status. Moreover, the lower use of print media by parents whose child has a disability implies unavailability or unsuitability of print information. The underlying reasons and information needs should be investigated further in order to ensure adequate information for parents with disabled children. High-quality information is relevant to all parents, irrespective of child’s health status, as they resort to the Internet frequently and specifically when paediatric visits did not answer all questions or raised new ones. Currently, digital health information for parents is largely offered by non-medical providers. The potential of digital media for parental health literacy could be increased if the demand for digital information were addressed by paediatric and public health professionals in a participatory approach.

## Supplementary Information

**Additional file 1: Table 1**. Comparison of sample characteristics between online and paper questionnaire.

## Data Availability

The dataset supporting the conclusions of this article and the questionnaire used for the survey are available in the Dataverse repository of Harvard University [10.7910/DVN/JI9GIJ].

## Abbreviations

BASEC: Business Administration System for Ethics Committees
CHF: Swiss Franca (Currency)
eHeals: Electronic Health Literacy Scale
SWIFS: Swiss infant feeding Study
