# Supplementary material for: Parents’ health information seeking behaviour – does the child’s health status play a role?
Source: BMC Fam Pract. 2020 Dec 10;21:266. doi: 10.1186/s12875-020-01342-3 (PMC7730778; doi:10.1186/s12875-020-01342-3)
Supplement: Supplementary file 1 — Additional file 1: Table 1. Comparison of sample characteristics between online and paper questionnaire. [file 12875_2020_1342_MOESM1_ESM.docx]

**Parents’ health information seeking behaviour – does the child’s health status play a role?**

Isabel Baumann, Rebecca Jaks, Dominik Robin, Sibylle Juvalta, Julia Dratva

**Table 1**: Comparison of sample characteristics between online and paper questionnaire

|  | **Questionnaire** | | **P-value** |
| --- | --- | --- | --- |
|  | Online | Paper |  |
|  | n=429 (56%) | n=430 (44%) |  |
| Use of digital media for general health and development | 392 (91.4) | 308 (90.6) | 0.704 |
| Use of digital media for acute child’s illness | 337 (81.4) | 273 (80.8) | 0.826 |
| No disability | 404 (94.2) | 318 (94.9) | 0.651 |
| Respondent’s characteristics |  |  |  |
| Age | 37.58 | 36.88 | 0.045 |
| Mothers | 365 (85.1) | 312 (92.9) | 0.001 |
| Tertiary education | 341 (79.5) | 239 (71.1) | 0.027 |

Note: The p-value indicates the result from paired-samples t-tests (age) and chi2-tests (all other characteristics).
